# Comparative Analysis of F-18 FDG PET-Based Metabolic Parameters in the Differential Diagnosis of Esophageal Squamous Cell Carcinoma and Adenocarcinoma

**DOI:** 10.1055/s-0046-1824603

**Published:** 2026-06-23

**Authors:** Priyank Rajput, Deepanksha Datta, Rajesh Kumar

**Affiliations:** 1 Department of Nuclear Medicine, Post Graduate Institute of Medical Education and Research, Chandigarh, India; 2 Department of Nuclear Medicine, All India Institute of Medical Sciences, Jodhpur, Rajasthan, India

**Keywords:** FDG PET, esophagus, PET-based radiomics, TLG, SUVmax

## Abstract

**Purpose:**

Intratumoral heterogeneity is a primary driver of treatment resistance in many malignancies, including esophageal cancer. While Fluorine-18 Fluorodeoxyglucose positron emission tomography/computed tomography (F-18 FDG PET/CT) is a standard tool for staging and response assessment in the esophageal malignancy, the qualitative visual assessment of primary tumor and metastases is limited by interobserver variability. The purpose of this study is to determine if the non-invasive imaging biomarkers on F-18 FDG PET can reliably distinguish the two primary histological subtypes of esophageal malignancy namely squamous cell carcinoma (SCC) and adenocarcinoma (AC).

**Methods:**

This is a retrospective study approved by the institutional ethical committee, and conducted in histopathologically proven cases of esophageal malignancy of either squamous cell or AC subtypes, who underwent baseline staging F-18 FDG PET/CT as per the standard guidelines. The metabolic parameters namely tumoral maximum standardized uptake value (SUVmax), total lesional glycolysis (TLG), metabolic tumor volume (MTV) and metabolic ratio of tumor to liver (standardized uptake ratio [SUR]) were calculated and compared between the two histological subtypes using Mann − Whitney U test. A *p* -value of less than 0.05 was considered statistically significant.

**Results:**

Out of 59 patients (M:F = 33:26) included in the study, 40 had squamous cell subtype and 19 had AC. Median age was 57 years (range: 30–83), and the median SUVmax, SUR, MTV, and TLG of AC group was 10.89 g/mL, 3.86, 9.98 mL, and 55.8 g/mL, while that of squamous group was 13.88 g/mL, 5.6, 13.52 mL, and 108.4 g/mL, respectively. There was significant difference noted in the tumoral SUVmax ( *p* = 0 *.043)* , SUR ( *p* = 0 *.019* ), and TLG ( *p* = 0 *.032* ) between the two groups, with higher metabolic values observed in the squamous group. MTV ( *p* = 0 *.224* ) between the two groups was insignificant.

**Conclusion:**

This study demonstrates that quantitative parameters on F-18 FDG PET are effective non-invasive biomarkers for differentiating the histological subtypes of esophageal malignancy. The squamous cell subtype exhibits significantly higher metabolism and total lesion glycolysis than AC.

## Introduction

Esophageal cancer is the eleventh most commonly diagnosed cancer and the seventh leading cause of cancer death worldwide. 1 In 2022, there were an estimated 510,716 new cases of esophageal cancer and 445,129 deaths globally. 2 3 The disease shows a marked gender disparity, with incidence and mortality rates being twofold to threefold higher in men than in women. 1 The geographic distribution is highly variable across different world regions with highest rates concentrated in Eastern Asia and Eastern Africa. Asia bears the heaviest burden, accounting for nearly 80% of all new cases. China alone accounts for more than half of the world's new cases. Incidence is significantly higher in less developed regions, where almost 80% of all cases occur. However, the incidence of the adenocarcinoma (AC) subtype is rising in high-income Western countries. India reported approximately 63,180 new cases in 2020, ranking second globally behind China in terms of estimated new cases. It is the fourth most common cause of cancer-related deaths in India. 4 Significant variation exists within the country. The Northeast region (Assam, Meghalaya, Mizoram, and Nagaland) and the Kashmir Valley are identified as high-incidence areas. In the Indian subcontinent, squamous cell carcinoma (SCC) is currently the most common type of esophageal cancer, with the distal third of the esophagus being the most frequently affected site. 5

Risk factors for esophageal cancer vary considerably between two main histological subtypes: SCC and AC. In high-income or transitioned settings, SCC is primarily linked to smoking and alcohol consumption, while in low-income or transitioning regions, although SCC remains highly prevalent, many of its specific risk factors are still being investigated. 6 7 Local traditional practices also play a significant role, with habits such as drinking very hot beverages, like salted tea in Kashmir, and poor oral hygiene contributing to the high incidence of SCC in certain “cancer belt” regions. In contrast, AC is strongly associated with lifestyle and metabolic factors, particularly excess body weight, which is expected to drive much of the future global disease burden. Clinical conditions such as chronic gastroesophageal reflux disease and Barrett's esophagus serve as major precursors to AC. 8 9 Furthermore, AC has become the dominant subtype in higher Human Development Index settings, accounting for about two-thirds of cases, and its incidence continues to rise in these populations. 10

18F-FDG PET/CT plays a critical role in the management of esophageal cancer by providing essential information for primary staging, therapy response assessment, prognostic stratification, and detection of recurrence. Compared with conventional staging investigations, PET/CT provides incremental data that changes the clinical management of approximately one-third of patients, primarily by identifying previously undetected distant metastases. 11 12 While its sensitivity for regional lymph node metastasis is considered moderate to low, its high specificity makes it a valuable tool for confirming nodal involvement. 13 In the context of neoadjuvant therapy, PET/CT can predict treatment response early in the course of chemotherapy or radiochemotherapy; a reduction in the standardized uptake value (SUV) by at least 35% is frequently used as a validated threshold to distinguish responders from non-responders. 14 Furthermore, the pre-treatment SUVmax of the primary tumor serves as a powerful prognostic marker, as higher metabolic activity is significantly associated with aggressive pathological features, such as deeper tumor invasion, and indicates a higher risk of recurrence and worse overall survival. 11 15 Finally, PET/CT is highly sensitive (98.4%) in the restaging setting for detecting suspected recurrence after curative-intent surgery, often identifying distant disease that shifts management from radical salvage treatment to palliative care. 16

Currently, F-18 FDG PET-CT provides the high-signal intensity required for reliable tumor segmentation and the extraction of metabolic radiomic features that predict survival and treatment resistance. These signatures identify high-risk patients who may not benefit from standard chemoradiation, especially when integrated with body composition data derived from PET/CT scan. However, the available literature on PET-based radiomics remains limited, particularly when it comes to differentiating the histological subtypes of esophageal carcinoma in baseline PET/CT imaging. In this study, our objective is to evaluate the role of FDG PET/CT in differentiating between the SCC and AC variants of esophageal cancer.

## Materials and Methods

### Study Settings

This study was conducted in the Department of Nuclear Medicine at our institute from February 2021 to March 2024. The study protocol was approved by the Institutional Ethical Committee (IEC number with date). Patient consent was waived off due to its retrospective nature.

### Inclusion and Exclusion Criteria

Patients with esophageal malignancy who had undergone upper gastrointestinal endoscopy with histopathological confirmation of either SCC or AC, and who had not received prior chemotherapy or radiotherapy, were included in this study. Exclusion criteria comprised patients with a history of chemotherapy, radiotherapy, or surgery for the **esophageal malignancy** , as well as those unwilling to undergo PET/CT, and pregnant or lactating women.

### F-18 FDG PET/CT Acquisition

Fluorine-18 Fluorodeoxyglucose Positron Emission Tomography/Computed Tomography (F-18 FDG PET/CT) imaging was performed in accordance with the standard clinical PET protocol as per the European Association of Nuclear Medicine guidelines. 17 The patients were intravenously injected with F-18 FDG 3.7 MBq/kg body weight to a maximum dose of 370 MBq after a 4 to 6 hour fasting period. All patients were imaged with an integrated PET-CT system (Discovery GE MIDR710). After 45 to 60 minutes of uptake period at rest, in a dimly lit quiet room, the images were acquired at 1 minute per bed position. In the patients with serum creatinine under normal limits and with no other contraindications to iodinated contrast, the PET scan was acquired together with the CECT scan, a delay of 70 seconds was between the intravenous iodinated contrast injection and acquisition of CT scan.

### Metabolic Parameters for Assessment

Two nuclear medicine physicians, each with over 8 years of experience, independently reviewed the PET/CT scans while remaining blinded to the histological subtypes. The metabolic parameters including tumoral SUV max, total lesion glycolysis (TLG), and metabolic tumor volume (MTV) were calculated using an automated region of interest (ROI) encompassing the entire metabolically active area. The standardized uptake ratio of tumor to liver (SUR) was determined by dividing the maximum standardized uptake value (SUVmax) of the primary tumor by the SUVmax of the liver. For this calculation, the SUVmax of the primary tumor was obtained from the automated ROI placed over the lesion, while the SUVmax of the liver was derived from the automated ROI positioned in the caudate lobe. This approach ensured consistency and reproducibility in the measurement of metabolic activity across patients.

### Statistical Analysis

The data was entered in Microsoft Excel spreadsheet (Microsoft technologies, United States). Continuous data was expressed as median (interquartile range) while discrete data was expressed as proportions. The statistical package for social sciences (SPSS) software (version 25) was used for all the statistical analysis. Inter-group comparisons were done using the Mann − Whitney U test. A *p* -value of less than 0.05 was considered statistically significant.

## Results

**Fig. 1:**
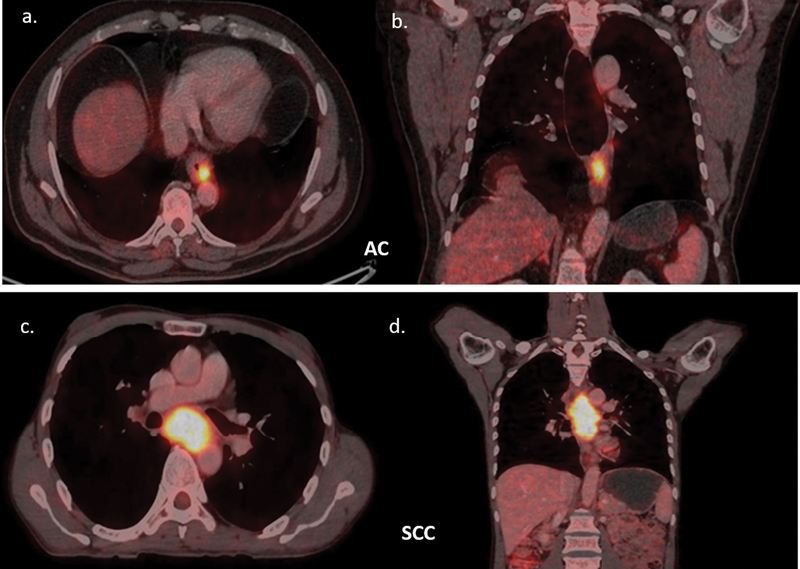
Representation images of each case of adenocarcinoma (AC) and squamous cell carcinoma (SCC) of esophagus.

Fifty-nine patients were included in this study, of which 33 were males (56%). Majority of the patients has SCC (40/59, 68%), and around one-third patients had AC (19/59). Fig. 1 shows the representation images of each case of SCC and AC.

**Fig. 2:**
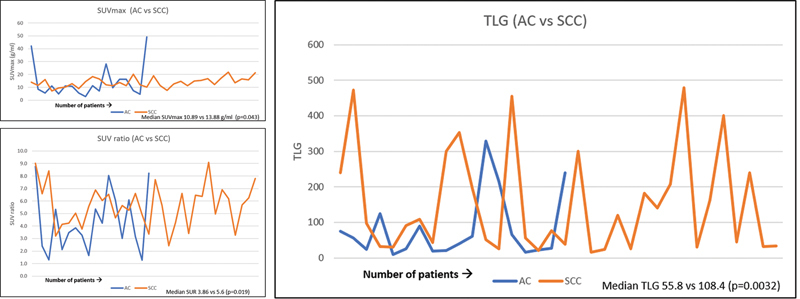
Comparison between the metabolic parameters between the adenocarcinoma (AC) and squamous cell carcinoma (SCC).

In the squamous subtype, the median values of SUVmax, SUR, MTV, and TLG were 13.88 g/mL, 5.6, 13.52 mL, and 108.4 g/mL, respectively. The AC subtypes showed the median values of SUVmax, SUR, MTV, and TLG as 10.89 g/mL, 3.86, 9.98 mL, and 55.8 g/mL, respectively. Statistical analysis revealed significant differences in SUVmax ( *p* = 0.043), SUR ( *p* = 0.019), and TLG ( *p* = 0.032), all favoring higher metabolic activity in SCC as shown in Fig. 2 . However, the difference in MTV between the two groups was not statistically significant ( *p* = 0.224). These findings suggest that SCC tends to exhibit greater metabolic aggressiveness compared with AC, independent of tumor volume, which may have implications for prognosis and therapeutic response assessment.

## Discussion

Despite advances in multimodality treatment, the prognosis for esophageal cancer remains poor. Accurate prediction of patient outcomes and survival in the preoperative setting is crucial, as it can guide the selection of personalized neoadjuvant or adjuvant treatment strategies. In this context, metabolic imaging plays a central role, not only in staging the disease but also in providing valuable predictive and prognostic information that can support more tailored approaches to patient management.

Esophageal SCC and AC are distinct biological entities despite arising in the same organ, differing in their cellular origins, molecular pathways, and genomic profiles. 18 SCC develops from squamous epithelial cells in the upper and middle esophagus, while AC originates from glandular cells in the lower esophagus, often linked to Barrett's esophagus. 19 Genomic studies, such as those from The Cancer Genome Atlas, show that SCC resembles head and neck SCC, whereas AC aligns more closely with the chromosomally unstable variant of gastric cancer. 20 Molecularly, SCC is characterized by amplifications of CCND1, SOX2, and TP63, with upregulation of WNT, SYN, and P63 pathways. Cyclin D1 amplification is notably more frequent in ESCC than in EAC. In contrast, AC demonstrates chromosomal instability, increased E-cadherin signaling, and HER2 overexpression in a subset of cases. Their mutational spectra also differ as SCC often shows indels and C:G > G:C transversions, while AC exhibits A:T > C:G transversions, particularly at the gastroesophageal junction. 21 Clinically, SCC is more prone to locoregional recurrence, while EAC more often leads to distant metastasis. Despite similar aggregate survival rates of 15 to 25%, the two subtypes differ in failure patterns, influencing staging, treatment, and prognosis. 22 At early stages (T1–T2, N0), their outcomes after curative resection are comparable. 23 In locally advanced disease, SCC responds better to neoadjuvant chemoradiotherapy, yielding superior locoregional control and survival. The landmark CROSS trial established neoadjuvant carboplatin–paclitaxel CRT followed by surgery as the standard for resectable esophageal cancer. 24 Subgroup analysis showed a far greater survival benefit in SCC (HR 0.42) than AC (HR 0.73), highlighting intrinsic differences in radiosensitivity. Chirieac et al further confirmed that pathological complete response after CRT is a stronger, independent predictor of long-term survival in SCC than in AC. 25 AC and SCC differ markedly in metastatic behavior, with direct prognostic implications. SCC tends to spread predictably to regional lymph nodes along the esophageal axis, with distant metastasis occurring later. In contrast, AC metastasizes earlier and more frequently, favoring the liver, lungs, peritoneum, and adrenal glands. 26 Rice et al further highlighted subtype-specific nodal patterns, with SCC exhibiting predictable drainage amenable to conventional radiation, whereas AC demonstrates variable nodal involvement, underscoring implications for radiation field design and lymphadenectomy. 27

Radiomics has shown potential in differentiating between AC and SCC, though its effectiveness depends on the imaging modality and clinical context. In F-18 FDG PET/CT scans, radiomics can detect significant textural differences between AC and SCC. 28 29 30 To date, studies using FDG PET/CT have primarily focused on prognostication and response assessment in esophageal cancer, while the use of PET-based radiomics to differentiate histological subtypes remains limited in the literature. In this study, we evaluated the role of PET-derived parameters in distinguishing between the two histological subtypes, thereby contributing to prognostication based on primary tissue histology. We observed notable differences in metabolic parameters between the SCC and AC groups. The SUVmax, TLG, and SUR were significantly higher in the SCC group than the AC group. Similar results were observed in the non-small cell lung cancer in which the SUVmax has consistently been reported as significantly higher in SCC than AC. Large-scale and smaller studies alike confirm this difference, with mean SUVmax values ranging from 8.85 ± 6.70 in SCC versus 5.87 ± 4.18 in AC, to 18.95 ± 8.3 in SCC versus 12.4 ± 7.55 in AC. 31 32 33 The biological basis for this disparity lies in several mechanisms. SCC demonstrates greater expression of glucose transporter type 1, which facilitates rapid glucose uptake, and higher levels of hexokinase 2, supporting increased metabolic activity for tumor growth. 34 35 Additionally, SCC exhibits faster proliferation rates, with a shorter tumor doubling time (92 days vs. 168 days in AC), and is more frequently associated with elevated HIF-1α expression, which enhances adaptation to hypoxic environments and correlates with higher SUVmax. 36 Together, these biological drivers explain the consistently greater metabolic activity observed in SCC than in AC.

The similarity in MTV between SCC and AC of the esophagus is largely explained by the fact that MTV reflects the physical size and longitudinal extension of the tumor rather than its histological subtype. Research shows that MTV values are directly proportional to the tumor's length and overall volume of hypermetabolic tissue, meaning both SCC and AC often present with comparable MTV when diagnosed at advanced stages such as T3 or T4. 37 The possible explanation for this could be that in locally advanced esophageal cancer, tumor volume tends to normalize across different histological subtypes because both SCC and AC follow similar growth patterns within the confined structure of the esophagus. This anatomical limitation results in comparable volumetric measurements, even though the tumors may differ in their biological behavior and metabolic intensity. Thus, while MTV does not significantly differentiate SCC from AC, other metabolic parameters provide more meaningful insights into their biological differences. However, our results were very different from Korkmaz et al in which only MTV showed an association between the SCC and AC, whereas clinical staging, TLG, and SUVmax did not demonstrate such a relationship. 38 This difference in the results could be mainly due to three reasons; first, out study cohort possibly exhibited more uniform tumor sizes across both histological subtypes, which may have minimized the volumetric differences reported by Korkmaz et al. Second, variation in tumor differentiation grades could also have played a role in our study. Poorly differentiated tumors, regardless of subtype, often demonstrate similar metabolic volumes. No information about tumor grades was mentioned in the baseline small-biopsy histopathology reports. Third, geographic and ethnic influences on tumor biology could also result in such differences, and future prospective multicenter studies are required to clarify them.

The retrospective design and a relatively small sample size are the major limitations of this study. In this study, we sought to address a gap in the existing literature by directly comparing various metabolic parameters between the two histological subgroups of esophageal cancer. While prior research has largely focused on prognostication and treatment response, limited attention has been given to evaluating how PET-based metrics differ across histologies. Our analysis aimed to explore these differences systematically, thereby contributing to a clearer understanding of the role of metabolic imaging in distinguishing between AC and SCC.

## Conclusion

Our findings demonstrate that SCC of the esophagus exhibits significant higher metabolic activity than AC of the esophagus, as evidenced by higher SUVmax, SUR, and TLG values. Importantly, these differences were independent of MTV, suggesting that metabolic aggressiveness of SCC is not merely a reflection of tumor size. This distinction may have prognostic relevance, highlighting the utility of PET-derived metabolic parameters in differentiating histological subtypes of esophageal malignancy.
